# High prevalence of respiratory symptoms among workers in the development section of a manually operated coal mine in a developing country: A cross sectional study

**DOI:** 10.1186/1471-2458-7-17

**Published:** 2007-02-01

**Authors:** Simon HD Mamuya, Magne Bråtveit, Yohana Mashalla, Bente E Moen

**Affiliations:** 1 Centre for International Health, University of Bergen, Norway; 2 Section for Occupational Medicine, University of Bergen, Norway; 3 Department of Community Health, Muhimbili University College of Health Sciences, Dar es Salaam, Tanzania; 4 Department of Physiology, Muhimbili University College of Health Sciences, Dar es Salaam, Tanzania

## Abstract

**Background:**

Few studies of miners have been carried out in African countries; most are from South Africa, where the working conditions are assumed to be better than in the rest of Africa. Several studies have focused on respiratory disorders among miners, but development workers responsible for creating underground road ways have not been studied explicitly. This is the first study assessing the associations between exposure to dust and quartz and respiratory symptoms among coal mine workers in a manually operated coal mine in Tanzania, focusing on development workers, as they have the highest exposure to coal dust.

**Methods:**

A cross-sectional study was carried out among 250 production workers from a coal mine. Interviews were performed using modified standardized questionnaires to elicit information on occupational history, demographics, smoking habits and acute and chronic respiratory symptoms. The relationships between current dust exposure as well as cumulative respirable dust and quartz and symptoms were studied by group comparisons as well as logistic regression.

**Results:**

Workers from the development group had the highest dust exposure, with arithmetic mean of 10.3 mg/m^3 ^for current respirable dust and 1.268 mg/m^3 ^for quartz. Analogous exposure results for mine workers were 0.66 mg/m^3 ^and 0.03 mg/m^3^, respectively; and for other development workers were 0.88 mg/m^3 ^and 0.10 mg/m^3^, respectively.

The workers from the development section had significantly higher prevalence of the acute symptoms of dry cough (45.7%), breathlessness (34.8%) and blocked nose (23.9%). In addition, development workers had significantly more chronic symptoms of breathlessness (17.0%) than the mine workers (6.4%) and the other production workers (2.4%). The highest decile of cumulative exposure to respirable dust was significantly associated with cough (OR = 2.91, 95% CI 1.06, 7.97) as were cumulative exposure to quartz and cough (OR = 2.87, CI 1.05, 7.88), compared with the reference consisting of the group of workers with the lowest quartile of the respective cumulative exposure.

**Conclusion:**

The development workers in a coal mine had more acute and chronic respiratory symptoms than the mine and the other production workers. In addition, there was an association between high cumulative coal dust and respiratory symptoms.

## Background

Respiratory diseases have a distinct role in the health of miners, with important implications for morbidity and mortality [1,2]. Respiratory symptoms may be early manifestations of acquired respiratory diseases, and examining such symptoms among miners can be helpful during health surveillance of these dust-exposed workers. Various studies from industrialized countries have documented the relationship between exposures to coal dust and increased respiratory symptoms. Both longitudinal and cross-sectional studies [3-8] have shown that symptoms of persistent cough and phlegm production, breathlessness and wheezing relate significantly with individual cumulative exposure to respirable mixed coal dust.

The British Pneumoconiosis Field Research among 30 000 miners showed that coal dust contributes to the development of respiratory symptoms at an early age [9]. The US Coal Mine Health and Safety Act in 1969 set the legal respirable mixed coal dust standard for coal mines in the United States at 3 mg/m^3^, with a reduction to 2 mg/m^3 ^in 1973. Despite these standards, studies in the United States showed statistically significant associations between cumulative exposure to respirable dust and respiratory symptoms for miners joining the industry after 1970 [10]. Henneberger & Attfield [7] showed a high prevalence of dyspnoea and wheezing for coal workers joining the industry in the United States before 1970. This study suggested that respiratory symptoms might provide an early warning related to prior exposure and might be followed by impairment in lung functioning.

Previous studies have examined respiratory symptoms in subgroups of miners such as coal face, maintenance, transport, maintenance and surface [11,12]; coal face, backbye and surface [12]; and coal face, face return and face end [13]. However, the development workers who create mining paths for miners to extract coal have not been studied explicitly. In our previous study[14], this group of workers was highest exposed to respirable dust and quartz, indicating a high risk of respiratory symptoms and disorders. More information about these workers is considered to be important for health efforts in the mines, in order to avoid future respiratory disorders due to dust exposure. In developing countries, and specifically among workers in labor-intensive coal mines, few studies have investigated the relationships between respiratory symptoms and coal mine dust.

The purpose of this study is to assess the occurrence of acute and chronic symptoms and associations between symptoms and exposure to respirable dust and quartz among coal mine workers in this manually operated coal mine in Tanzania, with a special focus on the development workers.

## Methods

### Study population

A cross-sectional study was carried out at a coal mine in Mbeya, Tanzania in 2003 and 2004. Of the 556 workers in this mine, 220 workers were excluded. The excluded workers were managers, assistant managers and heads of section due to their high socio-economic status. In addition surface workers in carpentry, masonry, garage, foundry, welding, machine workshop and surveying were excluded due to other types of dust exposure. Office workers and temporary workers were also excluded. Thus, 336 workers were invited to participate; 318 participated (303 men and 15 women), giving a response rate of 94.6%. The women were excluded before the statistical analysis due to their low number, as well as two workers with bronchial asthma and two with tuberculosis. The remaining 250 workers from the production part of the mine constituted the final study population. These were high-exposure workers from the development team (*n *= 47) and lower-exposure workers from the mine team (*n *= 78) and from the other production teams (n = 125). The tasks for the above teams are described in our previous publication (19).

### Questionnaire

The coal mine workers were interviewed using a respiratory health questionnaire. The questionnaire had three parts, including personal and work characteristics, smoking habits and respiratory health symptoms. The questionnaire was prepared in English and was translated into Swahili, the national language of Tanzania, it was used in the previous study[15]. The questionnaire was pre-tested among 30 selected coal mine workers and discussed for clarity before the study started. The questions on personal and work characteristics included sex, age, education level, employment history, years worked in the mine and years in dusty work elsewhere. The questionnaire was administered between 0800 and 1600.

Acute symptoms were assessed using a modified optimal symptom score questionnaire [16] and scored on a five-point Likert scale as never (1), mild (2), moderate (3), severe (4) or very severe (5). Workers were asked whether they had the following symptoms: dry cough, shortness of breath, wheezing, stuffy nose, runny nose and sneezing during or after the previous shift. Before statistical analysis, the responses were dichotomized to no (never) and yes (mild, moderate, severe or very severe).

A modified version of the British Medical Research Council questionnaire on respiratory symptoms [17] included questions on whether respondents usually had symptoms of cough, breathlessness and wheezing. The subjects were also asked whether they had bronchial asthma and/or other chronic illnesses such as tuberculosis and bronchitis (yes/no). Further, the workers were asked whether they had had injuries or surgery affecting the chest and whether they had had heart problems, pneumonia, pleurisy, pulmonary tuberculosis, bronchial asthma or any other chest problems in the past 3 years (yes/no). Those with any of these problems were excluded from the analysis.

Current smokers were defined as those who were smoking at the time of the study or those who had smoked more than one cigarette per day and stopped less than 1 year prior to the study. Ex-smokers were those who had smoked previously and stopped more than 1 year previously. The year they stopped smoking and the numbers of cigarettes smoked per day were also recorded. Never-smokers were defined as individuals who had never smoked.

### Assessment of exposure

As part of our previous exposure assessment [14], carried out concomitantly with the presently reported questionnaire studies on respiratory symptoms, personal dust was sampled during the day shift, which normally lasted about 5–10 hours. Five full-shift samples were taken on each monitoring day. Personal respirable dust was sampled using a SKC Sidekick pump (model 224–50) with a flow rate of 2.2 l/min. A rotameter was used to adjust the flow. The respirable dust samples were collected on 37-mm cellulose acetate filters (pore size 0.8 μm) placed in a 37-mm conductive plastic cyclone. The cassette was assembled and labeled at X-lab in Bergen, Norway. The cyclone was clipped to the worker's collar, allowing it to hang freely and collect dust in the breathing zone.

The respirable dust samples were quantified by gravimetric analysis using a Mettler AT 261 delta range with a limit of detection of 0.01 mg/m^3^. Respirable dust samples were analysed for quartz by X-ray diffraction on a silver membrane filter using NIOSH method 7500 at SGAB Analytica Laboratory, Luleå, Sweden. The limit of detection was 0.005 mg/m^3 ^[18].

### Cumulative dust exposure

The individual cumulative exposure to respirable dust or quartz (mg·year/m^3^) for the workers was estimated as the sum of the product of the estimated worker-specific mean exposure in the respective job teams and number of years the worker had spent in these job teams [19].

### Statistical analysis

The Statistical Package for the Social Sciences (SPSS) version 12 was used for the data analysis. *P *≤ 0.05 was chosen as the criterion for statistical significance. The independent *t*-test was used to compare continuous variables between the development, the mine and the other production workers. The chi-square test was used to compare proportions in categorical variables. Logistic regression analysis was used for groups where the number of workers with symptoms are about 15 [20] to determine odds ratio (OR) for groups with chronic respiratory symptoms based on quartiles and the highest deciles of cumulative exposure using the lowest quartile as the reference group, while adjusting for ever-smoking and age.

Summary variables for chronic symptoms and for acute symptoms were created to evaluate the correlation between chronic and acute respiratory symptoms. For chronic respiratory symptoms this was created by summarizing the score of each symptom; to have cough first thing in the morning, cough during the day and night, cough with sputum first thing in the morning, cough with sputum during the day and night, shortness of breath when hurrying on level ground and shortness of breath walking with people of your own age on level ground. This sum score ranged from 0 to 6. Summarizing the scores for dry cough, shortness of breath, wheezing, stuffy nose, runny nose and sneezing, created the summary variable of the acute respiratory symptoms with score (0–5). Pearson correlation coefficients were calculated for estimating the correlation between acute and chronic symptoms.

### Ethical approval and informed consent

Ethical approval was obtained from the Western Norway Regional Committee for Medical Research Ethics and the National Institute for Medical Research of Tanzania. The research permit was obtained from the Tanzania Commission for Science and Technology (COSTECH). There was institutional consent, since the administration of the Kiwira Coal Mine was informed of the project and allowed the study to proceed. Each person was informed about the aims of the study and the methods before being requested to consent to participate in the study voluntarily.

## Results

Table 1 shows the demographic characteristics and current and cumulative exposure to respirable dust and quartz. The arithmetic mean respirable dust and quartz exposure values were 18 and 12 times higher (respectively) for the development workers than for the mine workers, and 42 and13 times higher (respectively) for the development workers than for the group of other production workers. The cumulative exposure was also considerably higher for the development workers. The prevalence of current smokers and ever-smokers was not significantly different between the three groups. The number of years in the mine was significantly higher for other production workers than for mine workers. Further, the groups did not differ significantly in age, education or height (Table 1).

**Table 1:** Demographic characteristics, current and cumulative exposures to respirable dust and quartz among male workers in the coal mine

Exposure status	N	Age (years)^a^	Height (cm)^a^	Tenure (yrs)^a^	Ever smoker^b^	Current smoker^b^	Primary education only^b^	Current Dust^a ^(mg/m^3^)	Current Quartz^a ^(mg/m^3^)	Cumulative dust exposure^a ^(mg·yr/m^3^)	Cumulative quartz exposure^a ^(mg·yr/m^3^)
Development	47	36.1(9.6)	166.0(6.3)	9.3(6.9)	7(15.2)	4(8.7)	43(91.5)	10.3 (16.3)	1.27(3.40)	136.3(129.0)	6. 7(6.3)
Mine	78	36.1(6.5)	165.4(6.4)	9.4(5.3)	24(30.8)	6(7.7)	71(91.0)	0.66(0.61)	0.03(0.10)	23.5(48.8)	1.2(2.4)
Other production workers	125	36.9(6.9)	163.9(6.5)	11.5(5.1)	30(24.0)	13(10.4)	108(86.4)	0.88(1.6)	0.10(0.46)	24.5(51.4)	1.5(2.6)
All	250	36.5(7.3)	164.7(6.4)	10.4(5.6)	61(24.5)	23(9.2)	222(88.8)	3.7(9.97)	0.48(2.06)	45.2(84.0)	2.4(4.1)
P		0.714^c^	0.098^c^	0.008^c^	0.148^d^	0.803^d^	0.752^d^	<0.0001^c^	<0.002^c^	<0.0001^c^	<0.0001^c^

*n*: number of workers. ^a^Arithmetic mean (standard deviation). ^b^Number (percentage). ^c^Analysis of variance. ^d^Chi-square.

Workers in development, mine and other production differed significantly in the acute symptoms of dry cough, breathlessness and blocked nose (Table 2). The development workers had the highest prevalence of these symptoms (Table 2). For dry cough there was a significant difference between development and mine workers (P = 0.022) and between mine and other production workers (P = 0.047), respectively (Table 2). For breathlessness the significant difference was between development and other production workers (P = 0.005), while for blocked nose the significant difference was found between development and mine workers (P = 0.011). Among never smokers there was a significant difference between development and other production workers for breathlessness (Table 2).

**Table 2:** Acute respiratory symptoms among development, mine and other production workers in the of Kiwira coal mine

Symptoms		Development workers	Mine workers	Other production workers	*P *^1^
Dry cough	Never smoking	17(43.69%)	16(29.6%)	36(37.9%)	0.365
	All	21 (45.7%)	20(25.6%)	49 (39.2%)	0. 049
Breathlessness	Never smoking	12(30.8%)	9(16.7%)	14(14.7%)	0.087
	All	16 (34.8%)	13(16.7%)	19 (15.2%)	0.012
Blocked nose	Never smoking	6(15.4%)	5(9.3%)	15(15.8%)	0.514
	All	11 (23.9%)	6(7.7%)	17 (13.6%)	0.040
Running nose	Never smoking	19(48.7%)	27(50.0%)	53(55.8%)	0.681
	All	25 (54.3%)	37(47.4%)	71(56.8%)	0.425
Sneezing	Never smoking	13(33.3%)	40(42.1%)	40(40.3%)	0.607
	All	17 (37.0%)	29(37.2%)	51(40.8%)	0.835

^1^Chi-square test between the three groups of workers

For chronic symptoms the three groups of workers differed in cough as much as 4–6 times a day for 4 or more days a week and for breathlessness (Table 3). When only never-smokers were included in the analysis the three groups differed in cough with sputum production, cough as much as 4–6 times a day for 4 or more days a week and breathlessness (Table 3). The development workers had higher prevalence of breathlessness while walking with people of their own age than the group of other production workers (*P *= 0.002). This finding was persistent when including only the never-smokers stratification (*P *= 0.007). For cough as much as 4–6 times a day for 4 or more days a week the significant difference was between development and other production workers (*P *= 0.005 for all workers and *P *= 0.002 for never-smokers). There was no significant different between mine workers and the group of other production workers for any of the chronic symptoms.

**Table 3:** Chronic respiratory questions asked in a study of male coal miners comparing numbers and percentage of the affected among development, mine and other production workers stratified by smoking habit

Respiratory symptoms		Development workers n(%)	Mine workers n(%)	Others production workers0 n(%)	Total n(%)	*P*^1^
Do you usually cough first thing in the morning?	Never smoker	8(20.5%)	14(25.9%)	20(21.1%)	42(22.3%)	0.754
	All	10(21.3%)	21(26.9%)	29(23.2%)	60(24.0%)	0.741
Do you usually cough during the day or at night?	Never smoker	11(28.2%)	14(25.9%)	19(20.0%)	44(23.4%)	0.520
	All	13(27.7%)	21(26.9%)	28(22.4%)	63(25.2%)	0.593
*If the response was yes to any of the above, the worker was asked:*						

Do you usually cough as much as 4–6 times a day for 4 or more days in a week? (Yes/no)	Never smoker	8(20.5%)	6(11.1%)	4(4.2%)	18(9.6%)	0.013
	All	11(23.4%)	10(12.8%)	7(5.6%)	14(5.6%)	0.004
Have you coughed like this on most of days for as much as 3 consecutive months or more in a year? (Yes/no)	Never smoker	4(10.3%)	2(3.7%)	3(3.2%)	9(4.8%)	0.197
	All	5(10.6%)	5(6.4%)	4(3.2%)	14(5.6%)	0.156
*For cough with sputum production, a worker was asked:*						

Do you usually cough with sputum first thing in the morning? (Yes/no)	Never smoker	7(17.9%)	10(18.5%)	12(12.8%)	29(15.5%)	0.580
	All	9(19.1%)	15(19.2%)	18(14.3%)	42(16.9%)	0.614
Do you usually cough with sputum during the day or at night? (Yes/no)	Never smoker	7(17.9%)	8(14.8%)	8(8.4%)	23(12.2%)	0.246
	All	9(19.1%)	12(15.4%)	12(9.6%)	33(13.2%)	0.203
*If the response was yes to any of the above:*						

Do you usually cough with sputum as much as 4–6 times a day, or 4 or more days in a week? (Yes/no)	Never smoker	4(10.5%)	3(5.6%)	2(2.1%)	9(4.8%)	0.117
	All	4(8.7%)	6(7.7%)	5(4.0%)	15(6.0%)	0.393
Have you coughed with sputum on most of days for as much as 3 consecutive months or more in a year? (Yes/no)	Never smoker	3(7.7%)	1(1.9%)	0	4(2.1%)	0.019
	All	3(6.4%)	2(2.6%)	3(2.4%)	8(3.2%)	0.387
*Workers were classified as having breathlessness if they answered yes to:*						

Are you troubled by shortness of breath when hurrying on level ground? (Yes/no)	Never smoker	17(43.6%)	17(31.5%)	33(34.7%)	67(35.6%)	0.469
	All	21(44.7%)	25(32.1%)	40(32.0%)	86(34.4%)	0.258
Do you get shortness of breath walking with other people of your own age on level ground? (Yes/no)	Never smoker	7(17.9%)	2(3.7%)	3(3.2%)	12(6.4%)	0.004
	All	8(17.0%)	5(6.4%)	3(2.4%)	16(6.4%)	0.002
*If the response was yes to any of the above:*						

Do you have to stop for breathing when walking at your own pace on level ground? (Yes/no)	Never smoker	2(5.1%)	1(1.9%)	3(3.2)	6(3.2%)	0.675
	All	3(6.4%)	4(5.1%)	4(3.2%)	11(4.4%)	0.617
Have you experienced wheezing sound from your chest? (Yes/no)	Never smoker	5(12.8%)	4(7.4%)	5(5.4%)	14(7.5%)	0.335
	All	6(12.8%)	6(7.7%)	8(6.5%)	20(8.1%)	0.403

^1^Chi-square test between the three groups of workers

The workers in the highest decile of cumulative exposure to respirable dust and quartz had significantly higher odd ratios for chronic cough compared with the reference: 2.91 (1.06, 7.97) and 2.87 (1.05, 7.88), respectively (Table 4). Acute respiratory symptoms were highly correlated with the chronic respiratory symptoms (*r *= 0.400, *P *< 0.0001).

**Table 4:** Logistic regression of chronic respiratory symptoms and cumulative dust and quartz in quartiles and highest decile of cumulative exposure in a study of male coal miners adjusted for age and ever smoking.

Chronic symptoms	Exposure groups	Exposure (mg·years/m^3^)	*n*	No (%)	OR (95% CI)
Cumulative respirable dust					

Cough during the day or at night	First quartile	0.00–3.47	62	15 (24.2)	
	Second quartile	3.48–9.27	63	14 (22.2)	0.98 (0.43, 2.24)
	Third quartile	9.28–39.00	64	14 (21.9)	0.91 (0.39, 2.09)
	Fourth quartile	39.01–436.75	60	20 (33.3)	1.50 (0.68, 3.35)
	Highest decile	127.44–436.75	24	11 (45.8)	2.91 (1.06, 7.97)

Shortness of breath hurrying on level ground	First quartile	0.00–3.47	62	21 (33.9)	
	Second quartile	3.48–9.27	63	15 (23.8)	0.62 (0.28, 1.34)
	Third quartile	9.28–39.00	64	28 (43.8)	1.51 (0.74, 3.12)
	Fourth quartile	39.01–436.75	60	22 (36.7)	1.15 (0.55, 2.44)
	Highest decile	127.44–436.75	24	10 (40.0)	1.37 (0.52, 3.62)

Cumulative quartz					

Cough during the day or at night	First quartile	0.006–0.1615	62	15 (24.4)	
	Second quartile	0.162–0.432	64	15 (23.4)	0.88 (0.38, 2.04)
	Third quartile	0.433–2.825	61	14 (22.6)	0.88 (0.38, 2.02)
	Fourth quartile	2.826–21.372	62	19 (31.1)	1.61 (0.73, 3.58)
	Highest decile	6.232–21.372	25	11 (45.8)	2.87 (1.05, 7.88)

Shortness of breath hurrying on level ground	First quartile	0.006–0.1615	62	31 (33.9)	
	Second quartile	0.162–0.432	64	15 (23.4)	0.57 (0.26, 1.25)
	Third quartile	0.433–2.825	61	28 (45.9)	1.64 (0.79, 3.40)
	Fourth quartile	2.826–21.372	62	22 (35.5)	0.91 (0.42, 1.98)
	Highest decile	6.232–21.372	25	10 (40.0)	1.08 (0.33, 3.57)

## Discussion

The workers in the development section of the mine were significantly more affected by the acute symptoms of breathlessness and blocked nose compared with the other production workers. The higher exposure to respirable dust and quartz compared with other workers might explain this [14]. Our study also associated the presence of chronic respiratory symptoms and exposure to quartz and respirable coal mine dust. The fact that the specific group of workers from the development section has higher exposure and higher occurrence of symptoms has not been shown before. It might be that, awareness of high exposure is related to greater willingness to respond positively to questions about symptoms. However, in our study symptoms like runny nose and sneezing, not traditionally considered to be related to dust exposure were not different between the development, mine and the others and this strengthens our findings.

Our study showed a lower prevalence of chronic symptoms than previous studies from the United States, the United Kingdom and China. This might be explained by lower dust exposure levels in the present study. Workers in the mine team (coal face) had an average exposure of 0.66 mg/m^3 ^[14,19], which was lower than in previous studies in the United States (1.1 ± 0.5 mg/m^3^)[21], Australia (1.51 ± 1.08 mg/m^3^)[22] and South Africa (0.9 – 1.9 mg/m^3^)[23].

As a reminder, the frequency of chronic symptoms in the current study were 25.3% for any cough, 5.6% for chronic cough, 13.3% for any cough with sputum, 3.2% for chronic cough with sputum, 34.5% for short of breath when hurrying on level ground, and 8.1% for wheeze. The National Study of Coal Workers' Pneumoconiosis in the United States showed that 35% of the workers employed in coal mines before 1970 had chronic bronchitis (chronic cough and phlegm), 43% had shortness of breath and 42% had wheezing [7]. Seixas et al. [10] studied 1185 workers who started mining from 1970 and later; the prevalence of respiratory symptoms was lower, by reporting that 28% had cough, 32% phlegm, 21% chronic bronchitis, 22% breathlessness and 27% wheezing. Another study [11] among coal miners in the United States reported the prevalence of chronic bronchitis to be 33%, and studies of coal miners in the United Kingdom found that the prevalence of chronic bronchitis was 37% [5] and 39% [24]. A study of coal mine workers in China [8] showed that 77% had breathlessness walking at a normal pace on level ground, 47% had chronic cough and 37% had chronic phlegm.

The studies in the United Kingdom and the United States showed that chronic respiratory symptoms were associated with both smoking and dust exposure levels [5,11,24]. When converting gm hr/m^3 ^to mg- yrs/m^3 ^by using a factor of 1.74[4], the cumulative dust exposurefor coal miners of 250 gm-hr/m^3^reported by Rae et al. [24] is equivalent to 144 mg-yrs/m^3 ^which is close to 136.3 mg- yrs/m^3 ^presently found for development workers. Further, the mean age of the development workers of 36 years falls half way between the age groups of 25–34 and 35–44 described by Rae et al. [24]. For never-smokers in these two age groups, Rae et al. [24] reported an observed prevalence of cough with sputum for most days for 3 months of 20% and 22.2%. This is about 3 times greater than 7.7% reported by the development workers in the present study.

Kibelstis et al [11] showed that in each age group cigarette smoking coal face workers had significantly higher prevalence of respiratory symptoms than their non-smoking counterparts. In the study by Seixas and co-workers [10], never smokers had lower prevalence of respiratory symptoms than ex smokers and current smokers. However, the prevalence of respiratory symptoms in our study is lower than reported by Seixas et al [10] also among never-smokers.

The current prevalence of chronic cough of 5.6% is comparable to that reported by Naidoo et al. in South Africa (5.3%), who also reported relatively low prevalence of cough (9.0%), chronic phlegm (8.6%) and chronic bronchitis (7.5%) [23,25].

The prevalence of acute respiratory symptoms has to be interpreted with caution, as they correlate significantly with chronic symptoms. This may imply either that people with chronic symptoms also experience more acute symptoms or that people with chronic symptoms report the problem as an acute symptom. The definition of acute symptoms might confuse workers with chronic symptoms, thus exaggerating the acute respiratory problems among the coal mine workers.

The strengths of the current study include the availability of quantitative exposure data and the large contrast in exposure between the groups. However, we could only investigate relative differences in symptom prevalence between the exposed groups since we did not include an external group not exposed to mixed coal dust. The results indicate an association between dust exposure and respiratory symptoms, since stratification by smoking habits did not alter the significant difference in the prevalence of cough as much as 4–6 times a day for 4 days or more in a week and shortness of breath walking with people of own age between the groups; but a cross-sectional study cannot confirm causal relationships.

Further, information bias might have affected the reporting of symptoms. Our study took place when Tanzania was implementing public sector reform: moving from public ownership of industry into private or mixed public-private ownership. The planning of this process had started in the present mine at the time of our study and some workers were presumably afraid of losing their jobs because they could not be absorbed into the private sector immediately. In this context, some workers in the mine might not have given correct information on respiratory symptoms by thinking that such information could be used as a screening criterion to prevent future employment. This might have contributed to the low symptom prevalence observed in this study, although all workers were assured confidentiality during participation.

The healthy worker effect might also be an issue since only the current workers in the mine were studied. Workers who had developed respiratory symptoms and airflow limitation might have left the mining industry, thus contributing to underestimating the effect of exposure.

The use of respirable coal mine dust samples might be misleading, since the development of some of the respiratory symptoms might be more closely related to larger dust particles. However, Seixas et al. [26] addressed this issue and concluded that a respirable dust concentration is a sensible proxy for measuring larger particles. The exclusive use of current exposure data in the construction of cumulative exposure is a limitation of the study. However, according to the management the coal production was fairly stable for the past two decades and no major changes in the production processes had been done, indicating that the current data is representative for also the past exposures. The exposure levels were also similar in the two periods of sampling in this study.

This study was conducted in a mine in Tanzania, and the results may be difficult to generalize to other countries, although the information might be valid for the mines elsewhere with similar characteristics. However, the information obtained will be useful in improving the working conditions in the mine.

## Conclusion

This study, the first of its kind among miners in Tanzania, describes the relationship between coal mine dust and respiratory symptoms. The development workers had a greater risk of experiencing respiratory symptoms. This information is important for raising awareness among policy-makers and the workers and employers in the mine sector. It is also useful in setting priorities for prevention strategies.

## Competing interests

The author(s) declare that they have no competing interests.

## Authors' contributions

SHDM designed and conducted the study, performed statistical analysis, wrote the initial draft and revised the manuscripts after consultation with the other authors. MB, YM and BEM participated in designing the study and revising the manuscript. All authors have read and approved the final manuscript.

## Pre-publication history

The pre-publication history for this paper can be accessed here:


